# Dietary Structure and Cardiometabolic Risk Factors: A Comparative Analysis of Lingnan and Central Plains Regions in China Based on China Nutrition and Health Surveillance 2015–2017

**DOI:** 10.3390/nu17132173

**Published:** 2025-06-30

**Authors:** Weiyi Gong, Jiguo Zhang, Huijun Wang, Hongyun Fang, Jian Wen, Ping Gan, Panpan Huang, Jiaqi Li, Jiayu Lu, Qin Zhuo, Gangqiang Ding

**Affiliations:** 1 National Institute for Nutrition and Health, Chinese Center for Disease Control and Prevention, Beijing 100050, China; gongweiyi@ninh.chinacdc.cn (W.G.); zhangjg@ninh.chinacdc.cn (J.Z.); wanghj@ninh.chinacdc.cn (H.W.); fanghy@ninh.chinacdc.cn (H.F.); 2 NHC Key Laboratory of Public Nutrition and Health, Chinese Center for Disease Control and Prevention, Beijing 100050, China; 3 NHC Specialty Laboratory of Food Safety Risk Assessment and Standard Development, Guangdong Provincial Center for Disease Control and Prevention, Guangzhou 511430, China; wenjian19750726@126.com (J.W.); gzgp163@163.com (P.G.); huangpp5@mail2.sysu.edu.cn (P.H.); iamljqgirl@smu.edu.cn (J.L.); lujyyywe@163.com (J.L.)

**Keywords:** Lingnan region, dietary structure, obesity, hypertensive, diabetes mellitus, hyperlipidemia

## Abstract

**Background**: The Lingnan region is characterized by a hot and humid climate and abundant, diverse natural resources, while the Central Plains region experiences distinct four seasons and has a rich agricultural culture. Both regions possess unique dietary traditions and preferences. This study aims to investigate the differences in dietary structure between the Lingnan region (Guangdong, Guangxi, Hainan) and the Central Plains region (Shanxi, Shaanxi, Henan) and their impact on health. **Methods**: Using cross-sectional survey data from the 2015–2017 China National Nutrition and Health Survey, this study selected residents aged 18 and above as the research subjects. Generalized linear models were employed to analyze differences in the intake of various food groups between the two regions, while logistic regression models were used to examine regional differences in the prevalence of obesity, hypertension, diabetes mellitus, and hyperlipidemia. **Results**: A total of 14,484 adults were included in this study. Lingnan participants consumed significantly more rice products, red meat, poultry, seafood, and dark-colored vegetables, while Central Plains residents had higher intakes of wheat products, other cereals, soybeans, and eggs. Lingnan exhibited lower prevalence rates of obesity (8.6% vs. 18.1%), diabetes (7.6% vs. 9.8%), and hypertension (33.0% vs. 46.9%) compared to the Central Plains, with no significant difference in hyperlipidemia prevalence. Adjusted analyses confirmed that Lingnan residents had significantly reduced risks of obesity (OR = 0.431, 95% CI: 0.388–0.479), diabetes mellitus (OR = 0.841, 95% CI: 0.744–0.950), and hypertension (OR = 0.564, 95% CI: 0.523–0.608). **Conclusions**: The dietary structure in the Lingnan region plays a positive role in cardiometabolic health. Further analysis of the combined effects of different foods on health could provide a scientific basis for future nutrition and health management.

## 1. Introduction

Globally, the relationship between dietary patterns and health is a key focus of both academic research and public health initiatives [1,2,3]. Diet is a critical modifiable factor influencing health outcomes and is shaped by a variety of factors, including natural conditions, economic development, and cultural traditions. In China, regional dietary cultures vary significantly due to differences in climate, geography, and customs. These variations lead to diverse dietary structures and health outcomes across different regions [4,5,6,7]. With rapid economic development and changes in lifestyle, dietary patterns are also evolving, transitioning from traditional to more modernized diets [8,9,10]. Regional differences in diet, shaped by natural conditions, economic development, and culture, have created unique dietary traits and chronic disease trends [11,12].

Cardiometabolic diseases (CMD), including cardiovascular disease and diabetes, are major causes of morbidity and mortality among adults worldwide [13], and they also impose a substantial economic burden [14,15]. Cardiometabolic risk factors, such as hypertension (HTN), diabetes mellitus (DM), obesity (OB, and hyperlipidemia (HL), are major contributors to cardiovascular disease and metabolic syndrome worldwide [16,17], and they are rising rapidly [18,19]. These risk factors often cluster and synergistically increase the risk for cardiovascular morbidity and mortality [20,21]. Dietary intake is a critical modifiable factor influencing these risks by affecting blood pressure, glucose metabolism, lipid profiles, and energy balance. Diets high in saturated fats, refined sugars, and sodium promote adverse cardiometabolic risk factors, whereas diets rich in fruits, vegetables, whole grains, seafood, and unsaturated fats demonstrate protective associations [22,23,24,25,26].

In China, regional dietary differences significantly influence the prevalence and patterns of chronic diseases. For example, Southern China’s Lingnan region, characterized by a warm, humid climate and abundant natural resources, traditionally features a dietary pattern rich in vegetables, fruits, and seafood, with an emphasis on light flavors [27]. In contrast, central China’s grain-based agricultural economy results in dietary structures dominated by wheat, corn, and higher oil consumption [10,28]. These regional dietary differences influence the prevalence and patterns of cardiometabolic risk factors and chronic diseases such as HTN, DM, OB, and HL [10,28].

Previous studies have explored dietary patterns and their associations with chronic diseases in various Chinese populations [5,7,8,10,28]. However, few studies have directly compared the Lingnan and Central Plains regions in this regard, both of which have a long history of culinary culture in China. We specifically hypothesized that the traditional Lingnan dietary pattern may offer cardiometabolic protection, as evidenced by reduced disease burden compared to the Central Plains dietary structure. To test this hypothesis, this study analyzes distinctive dietary features of the Lingnan region (including Guangdong, Guangxi, and Hainan) and their associations with major cardiometabolic risk factors relative to the Central Plains region (including Henan, Shanxi, and Shaanxi), using data from the China Nutrition and Health Surveillance (CNHS) 2015–2017. By comparing regional dietary structure and disease prevalence, we aim to provide evidence-based insights for developing region-specific nutritional strategies to mitigate chronic disease burdens.

## 2. Materials and Methods

### 2.1. Data Collection and Samples

Data for this cross-sectional study were sourced from the CNHS 2015–2017, in which the 2015 China Chronic Disease and Nutrition Surveillance in adults employed a multistage stratified cluster sampling design. Survey locations were selected from monitoring sites across all 31 provinces, autonomous regions, and municipalities in mainland China; detailed sampling methods and inclusion/exclusion criteria have been published previously [29]. This analysis included adults aged ≥18 years and <80 years with complete survey data, excluding pregnant women, lactating mothers, and individuals with daily energy intake <500 kcal or >5000 kcal. A total of 14,484 participants were ultimately included: 7249 from the Lingnan region and 7235 from the Central Plains region. The study obtained ethical approval from the Ethics Committee of the Chinese Center for Disease Control and Prevention (Approval No. 201519-B), and written informed consent was obtained from all participants.

### 2.2. Basic Information Survey

Basic information (including sex, age, urban/rural area, geographical location, education level, household per capita income, physical activity level, smoking status and alcohol consumption) was collected from all participating households and individuals through face-to-face interviews.

### 2.3. Physical Examination

Participants underwent morning physical examinations after fasting. Measurements included height, weight, and blood pressure. To ensure quality, identical instruments were used by uniformly trained investigators following standardized protocols. The height was measured using a TZG stadiometer produced by Wuxi Weighing Apparatus Factory Co., Ltd., which is located in Wuxi, Jiangsu Province, China. The weight was determined with an electronic scale, model TANITA HD-390, manufactured by Dongguan Bailida Health Equipment Co., Ltd., based in Dongguan, Guangdong Province, China. The blood pressure was assessed using an electronic sphygmomanometer, model Omron HBP1300, from Omron Dalian Co., Ltd., situated in Dalian, Liaoning Province, China. Height and weight were measured twice (precision: 0.1 cm/kg), while blood pressure was measured three times at 1 min intervals (precision: 1 mmHg). Mean values were used for analysis.

### 2.4. Laboratory Test

Fasting venous blood (8 mL) was collected for assessment of fasting plasma glucose, glycated hemoglobin (HbA1c), total cholesterol, triglycerides, High-Density Lipoprotein (HDL-C) and Low-Density Lipoprotein (LDL-C). Within 0.5 to 1.0 h after collection, the plasma and serum were separated and aliquoted into cryovials as required. Subsequently, the processed blood samples were transported to the national laboratory for unified testing via a professional biological sample transportation agency, using a cold chain delivery system.

### 2.5. Dietary Assessment

Dietary assessment was conducted using a 3-day 24 h dietary recall method to record participants’ food consumption. We collected data for three consecutive working days, including two weekdays and one weekend day. Dietary records that were not for a full day were excluded. For analytical purposes, foods were categorized into 12 groups: rice products, wheat products, other cereals (including whole grains, legumes, and tubers), soybeans, dark-colored vegetables, light-colored vegetables, fruits, red meat, poultry, dairy products, eggs and seafood.

### 2.6. Definition of Obesity, Hypertension, Diabetes Mellitus and Hyperlipidemia

General OB is well-defined using body mass index (BMI), which was calculated as weight (kg) divided by height squared (m^2^). According to the Chinese criteria for adults, obesity was defined as a BMI of 28 kg/m^2^ or higher [30]. HTN was defined as systolic blood pressure (SBP) ≥ 140 mmHg, diastolic blood pressure (DBP) ≥ 90 mmHg, and/or use of antihypertensive drugs again in the past two weeks [31]. DM was diagnosed in participants with fasting plasma glucose (FPG) ≥ 7.0 mmol/L and/or glycated hemoglobin (HbA1c) ≥ 6.5% [32]. HL was identified in individuals with serum total cholesterol (TC) ≥ 6.2 mmol/L, triglycerides (TG) ≥ 2.3 mmol/L, low-density lipoprotein cholesterol (LDL-C) ≥ 4.1 mmol/L, and/or high-density lipoprotein cholesterol (HDL-C) ≤ 1.0 mmol/L [33]. Additionally, those diagnosed by a doctor with these conditions were also included.

### 2.7. Covariates

The variables used for multivariable adjustment in GLM and logistic regression analyses were defined as follows: (1) Age was categorized into: 18–44, 45–59, and ≥60 years. (2) Sex as male or female. (3) Residential area as urban or rural. (4) Household income per capita as: missing, low (lowest tertile), medium (middle tertile), and high (highest tertile). (5) Physical activity level as low, moderate, or high based on total weekly Metabolic Equivalent of Task (MET-min/week) and duration of physical activities [34]. (6) Educational attainment as: primary school or below, junior high school, or senior high school and above. (7) Smoking status as a current smoker (yes) or non-smoker (never smoked or former smoker). (8) Alcohol consumption as a current drinker (yes) or non-drinker (no).

### 2.8. Statistical Analysis

All statistical analyses for the full text were completed using SAS v. 9.4 (SAS Institute Inc., Cary, NC, USA). Continuous variables were described with the mean and standard deviation and compared across different groups using the Wilcoxon test. Categorical variables were presented as counts and proportions (%). Comparisons of categorical variables between different groups were performed using the chi-square test. Generalized linear models (GLMs) were employed to assess differences in various food intakes between the two regions, with results presented as estimates and 95% confidence intervals (95% CI). Additionally, multiple logistic regression models with appropriate adjustments were used to evaluate the differences in the prevalence of OB, HTN, DM, and HL between the two regions, with results presented as odds ratios (OR) and 95% CI. These models were adjusted for age, sex, residential area, per capita household income, physical activity level, education level, smoking status, alcohol consumption and energy, and all covariates were converted into dummy variables. For all analyses conducted, statistical significance was determined using a two-sided *p*-value, with a threshold of less than 0.05.

Testing interaction effects between the two regions and factors including sex (male/female), age (18 ≲ 45, 45 ≲ 60, ≥60 years), income (low, medium, high) and living area (urban/rural) was conducted via subgroup analysis. This was achieved by comparing differences between the two regions within fully adjusted logistic regression models.

## 3. Result

### 3.1. Characteristics of Participants

The analytic sample comprised 14,484 adults, with 7249 from Lingnan and 7235 from Central Plains. As shown in Table 1, Participants from Central Plains were older and more likely to reside in urban areas. Lingnan had higher proportions of high-income households and lower missing income data. Educational attainment differed significantly: Central Plains had more junior high school graduates, while senior high school completion rates were comparable. Health behaviors varied: Central Plains reported higher moderate physical activity but lower vigorous activity, with reduced smoking and alcohol consumption. Lingnan exhibited lower prevalence rates of OB (8.6% vs. 18.1%), DM (7.6% vs. 9.8%), and HTN (33.0% vs. 46.9%) compared to Central Plains. HL prevalence was similar between regions (38.7% vs. 38.6%)

### 3.2. Comparison of Food Intake Between the Two Regions in China

Table 2 displays adjusted mean differences in daily food intake between regions. Participants from Lingnan consumed significantly more rice products (adjusted mean difference [AMD]: +151.275 g/day, 95% CI: 148.660–153.889), red meat (AMD: +82.219 g/day, 95% CI: 80.330–84.107), poultry (AMD: +27.646 g/day, 95% CI: 26.562–28.730), seafood (AMD: +49.081 g/day, 95% CI: (47.328–50.833), dark-colored vegetables (AMD: +71.319 g/day, 95% CI: 68.571–74.068), and fruit (AMD: +4.024 g/day, 95% CI: 1.914–6.134) compared to Central Plains. Conversely, Central Plains showed higher consumption of wheat products (AMD: −186.827 g/day, 95% CI: −189.524 to −184.130), other cereals (AMD: −96.737 g/day, 95% CI: −99.671 to −93.805), soybeans (AMD: −5.574 g/day, 95% CI: −6.172 to −4.975), and eggs (AMD: −4.946 g/day, 95% CI: −5.749 to −4.143).

### 3.3. Comparison of the Prevalence of Obesity, Diabetes, Hypertension, and Hyperlipidemia Between the Two Regions in CHINA

As shown in Table 3, After adjusting for multiple variables including age, sex, residential area, household income, physical activity, education, energy intake, smoking, and alcohol consumption, Lingnan residents showed a lower prevalence of OB (OR = 0.431, 95% CI: 0.388–0.479), DM (OR = 0.841, 95% CI: 0.744–0.950), and HTN (OR = 0.564, 95% CI: 0.523–0.608). However, no significant association was observed for HL (OR = 1.055, 95% CI: 0.984–1.131).

### 3.4. Subgroup Analysis

The stratification by gender, age, urban–rural residence, and income status, as well as their interactions with region, are presented in Table 3. Stratified analyses revealed differential interaction effects across demographic factors. For gender, while no significant interactions were observed for DM (*p* = 0.720) or OB (*p* = 0.817), HTN showed a significant interaction (*p* = 0.001) with reduced risk in both genders in Lingnan. Conversely, HL exhibited strong gender interaction (*p* < 0.001), with males displaying higher risk and females lower risk in Lingnan versus Central Plains. Age demonstrated no significant interactions for any condition. Urban–rural residence significantly modified HTN (*p* = 0.020) and HL (*p* < 0.001) risks, with urban Lingnan residents showing lower HTN but higher HL risk. Income status showed no significant interactions except for a borderline HL interaction (*p* = 0.098), where higher-income individuals in Lingnan had elevated risk.

## 4. Discussion

This study reveals profound dietary divergence between Lingnan and Central Plains regions, with significant cardiometabolic implications. Lingnan’s diet—characterized by abundant rice, seafood, dark-colored vegetables, and fruits—may be associated with 16–57% (OR: 0.840–0.431) lower risks of OB, DM, and HTN compared to Central Plains’ wheat-dominated pattern. Notably, OB, DM, and HTN are not merely risk factors for cardiometabolic health; they are also anticipated to rank among the top contributors to the loss of healthy life years globally within the 20 leading risk factors projected for the year 2040 [35].

The Lingnan dietary culture can be traced back to the Qin and Han dynasties (221 BC–220 AD). During the Tang and Song dynasties, Guangzhou, as a pivotal hub of the Maritime Silk Road, developed a diverse culinary system in the Lingnan region, characterized by a rich variety of cooking methods [36]. In contrast to the dietary patterns of the Central Plains region, which primarily relies on wheat as a staple food and favors stewing and pickling (e.g., Shanxi noodles and Henan stewed noodles), the Lingnan diet exhibits three distinct features: (1) Balanced intake of animal and plant proteins: The consumption of seafood is significantly higher (+49.5 g/d compared with the Central Plains), providing high-quality *n*-3 fatty acids and selenium; (2) Rich in phytochemicals: The intake of dark-colored vegetables and fruits is abundant in flavonoids and vitamin C; (3) Low-sodium cooking philosophy: Cooking techniques such as steaming and blanching [27] are employed to preserve the original flavors of ingredients and reduce the reliance on salt.

When compared with other regional healthy diets in China, the Lingnan diet, characterized by its emphasis on plant-based foods and aquatic products, shares similarities with the Jiangnan diet. The Jiangnan diet is a traditional Chinese dietary pattern characterized by its emphasis on vegetables, soy products, freshwater fish, and whole grains [37]. Both the Jiangnan diet and the Lingnan diet emphasize the intake of abundant vegetables and aquatic products. Evidence from two dietary intervention trials has demonstrated that the Jiangnan diet is effective in achieving better glycemic control and weight management, as well as in improving cardiometabolic risk profiles [37,38]. When compared with globally recognized healthy dietary benchmarks, the Lingnan diet demonstrates significant parallels to both the Mediterranean diet and Dietary Approaches to Stop Hypertension (DASH) diet. The Mediterranean diet is the most well-known and researched dietary pattern worldwide, which is distinguished by a plant-based food predominance, with olive oil as the principal lipid source, moderate seafood consumption, and restricted red meat intake, complemented by abundant herbs and spices [39,40,41,42].

Similar to the Mediterranean pattern, the Lingnan diet prioritizes seafood, fruits, and vegetables as foundational components. Furthermore, it aligns with the DASH principle of low-sodium preparation through its traditional cooking methods that emphasize natural flavors over added salt [43]. The DASH diet emphasizes a balanced intake of fruits, vegetables, whole grains, lean proteins, and low-fat dairy, while reducing sodium, saturated fats, and added sugars to promote heart health and lower blood pressure [44]. Extensive research has firmly established the protective effects of both the Mediterranean and DASH diets against a spectrum of chronic diseases, encompassing diabetes, cardiovascular disorders, cancer, age-related conditions, and an overall reduction in mortality rates. For instance, the Mediterranean diet has been proven to be associated with weight loss, lower rates of incident diabetes and better glycemic control [45,46,47]. Data from a China Multi-Ethnic Cohort study, involving 83,081 participants from seven ethnic groups, showed that both the DASH diet and the Mediterranean diet are negatively associated with blood lipids [48].

The consumption of abundant rice, seafood, dark-colored vegetables, and fruits in the Lingnan diet is associated with a reduced risk of chronic diseases. Mechanistically, the Lingnan diet confers cardiometabolic protection through multi-pathway modulation. The anti-inflammatory and lipid-regulating properties of its key components warrant further discussion. High seafood-derived *n*-3 fatty acids, such as eicosapentaenoic acid (EPA) and docosahexaenoic acid (DHA) have been shown to elevate plasma concentrations, suppressing NF-κB activation and downstream pro-inflammatory cytokines [49]. This mechanism is crucial in mitigating inflammation, a key driver of many chronic diseases. Concurrently, flavonoids abundant in dark-colored vegetables, including quercetin and kaempferol, have been demonstrated to activate AMPK (AMP-activated protein kinase)-mediated fatty acid oxidation [50,51]. This activation plays a pivotal role in attenuating hepatic lipid accumulation, thereby potentially reducing the risk of metabolic disorders. Regarding glycemic control, resistant starch, particularly formed in cooled indica rice, functions by delaying intestinal glucose absorption. This delay is beneficial in reducing postprandial hyperglycemia [52], a critical factor in the management of DM and the prevention of its complications.

Additionally, the fiber-polysaccharide complexes found in fruits and vegetables have been shown to modulate gut microbiota composition. Specifically, they increase the population of Bifidobacteria, which in turn enhances the secretion of GLP-1 (glucagon-like peptide-1) and improves insulin sensitivity [53]. This regulation is essential for maintaining electrolyte balance and blood pressure, thereby reducing the risk of cardiovascular diseases. In terms of vascular homeostasis, potassium-rich produce like bananas and lychees is instrumental. They counteract sodium-induced hypertension by regulating renal Na⁺/K⁺-ATPase activity [54]. This regulation is essential for maintaining electrolyte balance and blood pressure, thereby reducing the risk of cardiovascular diseases. Collectively, these mechanisms highlight the multifaceted benefits of the Lingnan diet in promoting cardiometabolic health.

However, compared to the Chinese Dietary Guidelines (CDG) 2022 [55], the Lingnan diet exhibits notable strengths alongside critical nutritional gaps. The high seafood consumption in Lingnan (51.9 g/day) surpasses the CDG recommendations (≥40 g/day), providing cardioprotective EPA/DHA, offering beneficial cardioprotective effects through EPA and DHA intake. However, the consumption of other cereals and dairy products in Lingnan remains markedly insufficient. Specifically, there is a considerable shortfall relative to the CDG 2022 recommendations for whole grains and legumes (50–150 g/day), tubers (50–150 g/day), and dairy products (300 g/day). These disparities underscore the importance of maintaining Lingnan’s plant and seafood dietary foundation while integrating key priorities from the CDG 2022, such as increasing low-fat dairy intake and substituting some refined rice with whole grains such as barley and buckwheat.

This study, which delves into the dietary structure of the Lingnan region and its correlation with cardiometabolic risk factors, as compared to the Central Plains, offers significant contributions to our understanding of chronic disease management within the context of China Nutrition and Health Surveillance from 2015 to 2017. While our observational data suggest protective associations of the Lingnan dietary pattern, longitudinal studies are needed to confirm causality. Future research should continue to investigate the long-term impacts of regional dietary patterns on chronic disease outcomes and focus on developing effective public health strategies to promote healthier eating habits nationwide.

A key strength of this study is its systematic comparison of dietary patterns between two geographically and culturally distinct regions in China—the coastal Lingnan area and the inland Central Plains. This comparative approach provides novel insights into how region-specific dietary practices, shaped by historical traditions and local food systems, differentially influence cardiometabolic risk profiles. To our knowledge, no prior studies have directly compared dietary structures and associated cardiometabolic risk factors between the Lingnan and Central Plains regions of China. The study leverages a substantial dataset from the 2015–2017 China National Nutrition and Health Survey, which includes a diverse adult population, thus enhancing the external validity of the findings. However, this study has several limitations. Its cross-sectional design prevents the establishment of a direct causal relationship between diet and health outcomes. Additionally, the reliance on self-reported dietary data introduces the potential for information bias, as participants may not accurately recall or report their food consumption. The study’s focus on specific regions also limits the generalizability of the findings to other areas with different dietary habits and cultural contexts. Therefore, further research employing longitudinal designs and randomized controlled trials, alongside improved dietary assessment methods, is warranted to confirm and extend these results.

## 5. Conclusions

This study suggests that the traditional Lingnan dietary structure—characterized by high consumption of rice, seafood, dark-colored vegetables, and fruits—may confer cardiometabolic protection and is associated with lower prevalence of obesity, diabetes and hypertension compared to the Central Plains’ refined wheat-dominated diet. In conclusion, the findings of this study emphasize the need for tailored dietary interventions. By optimizing dietary patterns and addressing specific nutritional gaps, it is possible to mitigate chronic disease burdens in China.

## Figures and Tables

**Table 1:** Characteristics of participants in the Lingnan region and the Central Plains region.

Characteristics	Lingnan Region		Central Plains Region			
	N	R/%	N	R/%	χ^2^	*p*-Value
Gender					0.009	0.926
Male	3401	46.9	3400	47.0		
Female	3848	53.1	3835	53.0		
Age (years)					101.553	<0.0001
18 ≲ 45	2414	33.3	1857	25.7		
45 ≲ 60	2667	36.8	2954	40.8		
≥60	2168	29.9	2424	33.5		
Living area					49.871	<0.0001
Urban	2859	39.4	3273	45.2		
Rural	4390	60.6	3962	54.8		
Income					465.400	<0.0001
Missing	1774	24.5	2721	37.6		
Low	1718	23.7	1830	25.3		
Medium	1557	21.5	1426	19.7		
High	2200	30.3	1258	17.4		
Education					53.857	<0.0001
Primary school						
or below	3374	46.5	3015	41.7		
Junior middle						
school	2288	31.6	2691	37.2		
High school or						
higher	1587	21.9	1529	21.1		
Physical activity					74.127	<0.0001
Mild	1623	22.4	1867	25.8		
Moderate	1776	24.5	2043	28.2		
Severe	3850	53.1	3325	46.0		
Smoking					7.402	0.007
No	5264	72.6	5398	74.6		
Yes	1985	27.4	1837	25.4		
Drinking					53.741	<0.0001
No	4544	62.7	4954	68.5		
Yes	2705	37.3	2281	31.5		
DM					22.330	<0.0001
No	6699	92.4	6526	90.2		
Yes	550	7.6	709	9.8		
OB					282.398	<0.0001
No	6628	91.4	5929	82.0		
Yes	621	8.6	1306	18.1		
HTN					291.722	<0.0001
No	4860	67.0	3845	53.1		
Yes	2389	33.0	3390	46.9		
HL					0.002	0.965
No	4446	61.3	4440	61.4		
Yes	2803	38.7	2795	38.6		
Total	7249	100	7235	100		

**Table 2:** Comparison of food intake in the Lingnan region and the Central Plains region ($\bar{x}\pm s$).

Food Group	Lingnan Region	Central Plains Region	Adjusted Mean Difference (95%CI) *
Rice products	185.6 ± 103.6	32.3 ± 44.8	151.275 (148.660, 153.889)
Wheat products	24.0 ± 38.7	212.5 ± 109.3	−186.827 (−189.524, −184.130)
Other cereal	17.0 ± 44.1	111.7 ± 116.7	−96.737 (−99.671, −93.805)
Soybean	6.8 ± 14.9	12.3 ± 20.3	−5.574 (−6.172, −4.975)
Dark-colored vegetables	114.4 ± 101.7	44.3 ± 55.9	71.319 (68.571, 74.068)
Light-colored vegetables	160.2 ± 120.7	163.3 ± 119.3	−3.816 (−7.839, 0.207)
Fruit	31.6 ± 63.4	28.2 ± 67.2	4.024 (1.914, 6.134)
Red meat	108.5 ± 70.3	25.6 ± 39.4	82.219 (80.330,84.107)
Poultry	31.1 ± 43.5	3.2 ± 15.0	27.646 (26.562, 28.730)
Milk products	8.3 ± 39.1	14.3 ± 51.4	−5.441 (−6.926, −3.956)
Eggs	14.3 ± 22.1	19.1 ± 26.5	−4.946 (−5.749, −4.143)
Seafood	51.9 ± 72.9	2.4 ± 12.6	49.081 (47.328, 50.833)

* Model adjusted age, sex, residential area, per capita household income, physical activity level, education level, smoking status and alcohol consumption.

**Table 3:** Comparison of the prevalence of obesity, diabetes, hypertension, and hyperlipidemia in the Lingnan region and the Central Plains region.

Subgroup	DM		OB		HTN		HL	
	OR (95% CI) *	*p* for Interaction	OR (95% CI) *	*p* for Interaction	OR (95% CI) *	*p* for Interaction	OR (95% CI) *	*p* for Interaction
Central Plains	Reference		Reference		Reference		Reference	
Lingnan Region	0.841 (0.744, 0.950)		0.431 (0.388, 0.479)		0.564 (0.523, 0.608)		1.055 (0.984, 1.131)	
Gender		0.720		0.817		0.001		<0.001
Male	0.850 (0.710, 1.017)		0.429 (0.367, 0.502)		0.649 (0.584, 0.722)		1.171 (1.060, 1.294)	
Female	0.828 (0.699, 0.980)		0.427 (0.370, 0.493)		0.487 (0.437, 0.542)		0.944 (0.855, 1.044)	
Age (years)		0.112		0.083		0.254		0.233
18 ≲ 45	0.745 (0.525, 1.057)		0.388 (0.321, 0.470)		0.558 (0.468, 0.665)		1.012 (0.882, 1.161)	
45 ≲ 60	0.952 (0.782, 1.159)		0.487 (0.414, 0.574)		0.550 (0.492, 0.616)		1.104 (0.989, 1.233)	
≥60	0.802 (0.672, 0.957)		0.413 (0.338, 0.506)		0.587 (0.518, 0.666)		1.039 (0.918, 1.175)	
Living area		0.174		0.071		0.020		<0.001
Urban	0.673 (0.567, 0.799)		0.486 (0.418, 0.564)		0.563 (0.504, 0.629)		1.284 (1.154, 1.429)	
Rural	0.862 (0.727, 1.022)		0.390 (0.337, 0.452)		0.502 (0.458, 0.551)		0.851 (0.777, 0.933)	
Income		0.299		0.318		0.252		0.098
Low	0.716 (0.563, 0.911)		0.295 (0.233, 0.372)		0.475 (0.417, 0.541)		0.870 (0.766, 0.988)	
Medium	0.920 (0.723, 1.170)		0.432 (0.352, 0.530)		0.582 (0.505, 0.671)		1.063 (0.926, 1.220)	
High	0.702 (0.549, 0.898)		0.509 (0.412, 0.629)		0.575 (0.492, 0.672)		1.226 (1.055, 1.424)	

* Model adjusted age, sex, residential area, per capita household income, physical activity level, education level, energy, smoking status, and alcohol consumption. Subgroup analyses were adjusted for all variables other than the subgroup variable itself.

## Data Availability

The data are not permitted to be disclosed according to the National Institute for Nutrition and Health, Chinese Center for Disease Control and Prevention.
